# HIV vaccines: progress and promise

**DOI:** 10.1002/jia2.25828

**Published:** 2021-11-21

**Authors:** Margaret I. Johnston, Gabriella Scarlatti, Punnee Pitisutthithum, Linda‐Gail Bekker

**Affiliations:** ^1^ Independent Consultant Vancouver Canada; ^2^ Division of Immunology Transplantation and Infectious Diseases San Raffaele Scientific Institute Milan Italy; ^3^ Faculty of Tropical Medicine Mahidol University Nakhon Pathom Thailand; ^4^ The Desmond Tutu HIV Center Cape Town South Africa

**Keywords:** HIV, vaccines, prevention, collaborations, monoclonal antibodies, active immunization, passive immunization, structural biology, germline targeting

As the end of 2021 nears, the world continues to battle the COVID‐19 pandemic. SARS‐CoV‐2, the etiological agent of COVID‐19, has infected over 200 million individuals and claimed the lives of over 4 million of them (https://covid19.who.int/). COVID‐19 continues to periodically surge in many parts of the world. Even though highly effective vaccines were identified and granted emergency use authorization in 2020, most middle‐ and lower‐income countries have yet to obtain and distribute sufficient doses to afford large‐scale protection against new infections. Furthermore, viral variants that arise during replication threaten to diminish the protective efficacy of first‐generation COVID‐19 vaccines [1].

Unlike HIV, most, but not all, SARS‐CoV‐2‐infected individuals mount an effective immune response that leads to full recovery, which provided a “proof‐of‐concept” that an effective vaccine could be identified. Enormous financial incentives and novel funding mechanisms ensued [2]. Private companies readily took up the challenge and advanced candidate vaccines into human studies in record time. In many cases they employed platform technologies, such as recombinant adenovirus 26 and mRNA, that had been explored in the development of vaccines to prevent HIV, Ebola, influenza and other infectious diseases [3]. The scientific community, especially HIV vaccine trialists with established clinical sites, community relationships and laboratory procedures, quickly pivoted and formed new alliances to demonstrate the success of those vaccines [4].

Now, 40 years after the first report of the disease now known as AIDS, proof that an HIV vaccine is possible, has relied heavily on active and passive vaccine studies in non‐human primates (NHP), the RV144 phase 2b trial in Thailand, and studies of passively administered monoclonal antibodies in NHP and, more recently, humans [5, 6, 7, 8] The scientific challenges faced in the quest to identify a safe and effective HIV vaccine remain unchanged: enormous variability in the outer membrane envelope, which enables evasion from immune responses; a “glycan shield” that hides sites that might otherwise be vulnerable to antibody (Ab) neutralization, and the absence of financial incentives that would sway the private sector to put their full weight into the effort [9].

This special issue of the *Journal of the International AIDS Society* (JIAS) was conceived as an opportunity to take stock in progress on HIV research and development, outline novel scientific and organizational approaches that might lead to success, and learn from HIV and SARS‐CoV‐2 vaccine studies.

## 1 CURRENT PREVENTION TECHNOLOGIES ARE NOT LIKELY TO END AIDS

Efforts to identify HIV prevention approaches beyond barrier methods have resulted in several options for populations with access to HIV testing and antiretrovirals (ARVs). As summarized by Fauci et al. [10] in this issue, these options include treatment of persons with HIV to lower virus levels below what is required for transmission; oral, injectable or vaginal ring delivered pre‐exposure prophylaxis (PrEP) with potent ARVs that prevent the virus from establishing infection [10]. As with barrier methods, these interventions require consistent adherence, which is challenging in settings where stigma is prominent, or where dependable access to these interventions is not feasible due to logistical, cost or other obstacles. On‐going efforts to develop long‐acting ARVs or broadly neutralizing antibody (bNAb) cocktails for prevention, which if only required every 6–12 months, could somewhat ease these challenges.

Since all existing prevention methods require adherence, product developers and trialists need to design and evaluate approaches that individuals will effectively use. Acquiring community perspectives and input into product development and testing and understanding what individuals at risk are most likely to consistently use, are imperative to success. Given the wide diversity of at‐risk populations, including men who have sex with men, sex workers, people who use drugs, adolescent girls and young women in sub‐Saharan Africa, providing some choice in prevention interventions will likely be required to achieve population‐level impact on virus spread. As attested to by Luba et al. [11] in this issue, a safe and highly effective vaccine would be a valuable addition to the existing prevention toolbox.

## 2 NEW MODELS OF COLLABORATION AND PARTNERSHIP ARE PROVING FRUITFUL

Advances made in the past 25 years have been facilitated by the creation of new organizations and collaborations designed to bring additional resources and energy into the HIV vaccine development field. One such organization, International AIDS Vaccine Initiative (IAVI), has made significant contributions over the years, as described in Feinberg [12]. Perhaps most notably, IAVI researchers collected specimens that enabled isolation of broadly neutralizing antibodies and facilitated the transition of new candidates from academic settings into the clinic [9] leading to some very exciting new antibody candidates.

A significant shift in the field took place following the 2003 Science publication calling for creation of a Global HIV Vaccine Enterprise (the Enterprise), which received support from the G8 the following year [13, 14]. The G8 called for creation of an alliance of researchers from around the globe to synergize efforts, avoid unnecessary duplication of efforts, standardize laboratory measurements to permit valid comparisons among studies and promote greater capacity for the manufacture and distribution of any vaccine. This call to action inspired the formation of two new collaborative inter‐disciplinary programs that focus on advancing novel vaccine designs, that is, The Center for AIDS Vaccine Immunology, now the Center for AIDS Vaccine Development and the Collaboration for AIDS Vaccine Discovery (CAVD). Notably, CAVD researchers are required to share data with others in the CAVD program prior to publication, and CAVD awards include global access requirements. About the same time, the HIV Vaccine Trials Network was expanded to include more international sites, especially in Africa and South America, which has proven key to the successful conduct of efficacy studies for both HIV and SARS‐CoV‐2. The evolution of these and other important collaborative efforts, including EAVI2020 and Accelerate the Development of Vaccines and New Technologies to Combat the AIDS Epidemic (ADVANCE), are summarized in Feinberg et al. [12].

The Global HIV Vaccine Enterprise also evolved over time and has served to bring together stakeholders to discuss and address roadblocks such as how HIV vaccine efficacy trials might be conducted in the face of expanded access to other prevention approaches. Tatoud and co‐authors provide a valuable perspective on the past and potential future role of the Enterprise [15].

The UNAIDS has also made significant contributions to the field, most notably the creation of a consensus document on the ethical considerations in HIV prevention trials [16]. Almost all guidance points have changed as the prevention field has evolved; key changes are summarized in Slack et al. [17].

## 3 WHERE DO WE NOW STAND?

The lack of an effective vaccine has not been due to a lack of effort. Over $800 million USD was spent on HIV vaccine research and development in 2019 alone [18]. To date, one study conducted in Thailand showed modest vaccine efficacy, which appeared to wane over time [6, 19]. A modified version of that vaccine, when tested in a higher incidence population in South Africa, failed to show any efficacy [20]. Other advanced candidates have either failed to show efficacy or remain under study, as summarized in Kim et al. [7].

One novel vaccine design still in efficacy testing at the time of this writing is based on mosaic antigens, which were designed to afford broad coverage across HIV clades. The Imbokodo and Mosaico trials are evaluating a prime‐boost regimen that combines an rAd26 vector and protein. Unfortunately, following a recommendation from its data and safety monitoring board, the Imbokodo study will not be moving forward to its second phase of follow‐up due to inadequate efficacy although no safety concerns have been raised. Another study referred to as PrEPVacc is evaluating PrEP and two vaccine candidates: rDNA with gp120 boost and rDNA/gp140 followed by MVA/gp140 boost. This novel multi‐arm, multi‐stage adaptive trial will employ an averted infection ratio method to determine efficacy. These trials, other on‐going and past vaccine studies, as well as innovative studies of bNAbs, native envelope trimers, germline targeting immunogens, mRNA and new adjuvants are reviewed in Kim et al. [7]. Most recently, Moderna has launched a first in human safety trial of a novel mRNA‐based vaccine to prevent HIV (mRNA‐1644) in partnership with IAVI.

Vaccine design has benefited from improved structural biology tools, as reviewed by Derking et al. [21]. Structural studies have provided insights as to how to modify the HIV envelope to expose sites that are vulnerable to neutralization, as well as how to develop stable, “authentic” looking HIV envelope trimers. Understanding how the enormous glycan shield on the HIV envelope protein masks certain sites of vulnerability has led researchers to produce immunogens designed to induce antibodies that are “on target” and avoid inducing antibodies that are “off target.” Recent technical advances now enable the identification of the sites of antibody binding at an atomic level.

The sera of a small percentage of individuals living with HIV contains bNAbs, which has provided the proof‐of‐concept that a bNAb‐inducing vaccine is possible. Detailed studies of how both the HIV envelope and the body's Ab response evolve over time in those individuals have led to a strategy that attempts to replicate that evolutionary process through sequential immunization with multiple immunogens [22]. The first step along this pathway is to devise an immunogen that activates unmutated common ancestors of the desired bNAb, followed by boosting with different immunogens designed to “mature” the response to one that includes plasma cells producing the desired bNAb. This strategy and specific examples being pursued are reviewed in technical detail by Williams et al. [23].

Schief and collaborators designed a germline targeting immunogen, eOD‐GT8, that was able to bind to VRC01‐class precursor naïve B cells from uninfected individuals [24]. Subsequent studies initially described at the R4P conference in early 2021 demonstrated that this immunogen could expand such cells in human volunteers [25]. This is perhaps the most promising study to date supporting the hypothesis that vaccines might be able to do better than the immune responses and induce bNAbs that neutralize HIV upon its entry into the body, or that immune memory of such responses will be sufficiently fast and durable to prevent the establishment of infection. Now comes the challenge of determining what additional immunogen(s) are required to trigger further B‐cell development along the desired pathway, which could benefit from the application of mRNA vaccine technology used in COVID‐19 vaccines. In addition, responses will need to be more durable and broader than the CD4 binding site targeted by VRC01.

An alternative to the induction of bNAbs through active immunization is the passive administration of bNAbs, referred to as AMP, or antibody‐mediated protection. Two studies of a passively administered bNAb, VRC01, to volunteers at risk for HIV infection were completed recently. As summarized in Miner et al. [8], infusions of VRC01 administered every 8 weeks did not prevent infection overall, but VRC01 was highly effective at preventing infection with strains of HIV that were susceptible to VRC01 in vitro at the level of Ab achieved in the trial. This has led to the hypothesis that protection could be achieved following administration of mixtures of bNABs (or bivalent or trivalent bNAbs) that are sufficiently potent to neutralize a high percentage of viruses in vivo. Such studies are underway or planned. Importantly, the results of that initial VRC01 trial demonstrated that neutralization in the TZM‐bl/pseudovirus in vitro assay can reliably predict protection from HIV infection in humans, thus, setting the stage for a rationale selection of antibodies that could prove highly effective [24].

## 4 THE FUTURE

Despite the disappointment of Imbokodo the Mosaico efficacy study, which is being conducted in men who have sex with men and transgender people, and which employs a boost with two glycoproteins, is continuing. If Mosaico shows a modest or better level of efficacy, then hope remains that induction of antibodies that bind to but do not necessarily neutralize HIV, along with HIV‐specific T cells could prove beneficial. If the results are “modest” (i.e., lower bound of efficacy estimate above zero but not sufficiently high to warrant licensure, e.g., <70%), then efforts to improve upon those results will likely fall to the corporate sponsor, governments and non‐profit partners. Whether such a collaborative effort will be undertaken will likely depend on the progress made with other passive and active immunization strategies as well as the uptake of existing prevention measures. If, however, efficacy is sufficiently high to warrant licensure, then attention will need to turn to tackling implementation issues including bridging to other populations and other virus clades.

Similarly, if passive administration of bNAbs or active immunization with a series of immunogens, as proposed by Corey et al. [8] and Williams et al. [23], respectively, prove successful, practical downstream challenges will still need to be addressed. These include how to integrate those complex regimens with other prevention strategies such as oral PrEP and long‐acting PrEP; how cost‐effectiveness data will be considered; whether manufacturing and distribution systems are sufficient; and how public health agencies will take personal choice into consideration when deciding what interventions to support. These questions have important ramification for health care systems in both highly and under‐resourced settings. How best to obtain prequalification from the World Health Organization (WHO) and a positive recommendation of WHO's Strategic Advisory Group of Experts on Immunization, the role of Gavi, the Vaccine Alliance and gathering of real‐world effectiveness data will need to be tackled. These issues will require continued, novel collaborations among stakeholders, with special consideration to the views of the potential “user” community, as even the best prevention measures will fail if they are not only safe and effective, but also available, affordable, accessible and reliably utilized.

## COMPETING INTERESTS

The authors declare no competing interests.

## Authors’ contributions

MIJ prepared the initial draft; co‐authors provided editing and content revisions; all authors approved final draft.

## Disclaimer

LGB is on the Expert Advisory Group of the Global HIV Vaccine Enterprise.
